# Targeting latent viral infection in EBV-associated lymphomas

**DOI:** 10.3389/fimmu.2024.1342455

**Published:** 2024-02-23

**Authors:** Isabella Y. Kong, Lisa Giulino-Roth

**Affiliations:** Weill Cornell Medical College, New York, NY, United States

**Keywords:** ebv, lymphoma, latency, T-cell, EBNA2, EBNA3, LMP1

## Abstract

Epstein-Barr virus (EBV) contributes to the development of a significant subset of human lymphomas. As a herpes virus, EBV can transition between a lytic state which is required to establish infection and a latent state where a limited number of viral antigens are expressed which allows infected cells to escape immune surveillance. Three broad latency programs have been described which are defined by the expression of viral proteins RNA, with latency I being the most restrictive expressing only EBV nuclear antigen 1 (EBNA1) and EBV-encoded small RNAs (EBERs) and latency III expressing the full panel of latent viral genes including the latent membrane proteins 1 and 2 (LMP1/2), and EBNA 2, 3, and leader protein (LP) which induce a robust T-cell response. The therapeutic use of EBV-specific T-cells has advanced the treatment of EBV-associated lymphoma, however this approach is only effective against EBV-associated lymphomas that express the latency II or III program. Latency I tumors such as Burkitt lymphoma (BL) and a subset of diffuse large B-cell lymphomas (DLBCL) evade the host immune response to EBV and are resistant to EBV-specific T-cell therapies. Thus, strategies for inducing a switch from the latency I to the latency II or III program in EBV+ tumors are being investigated as mechanisms to sensitize tumors to T-cell mediated killing. Here, we review what is known about the establishment and regulation of latency in EBV infected B-cells, the role of EBV-specific T-cells in lymphoma, and strategies to convert latency I tumors to latency II/III.

## 1 Introduction

Epstein-Barr virus (EBV), also referred to as human herpesvirus 4 (HHV-4), is a lymphotropic gamma-herpes virus that infects over 95% of adults worldwide. EBV can transform B-cells *in-vitro* into immortal lymphoblastoid cell lines (LCLs). *In-vivo* EBV is associated with a range of malignancies including B-cell lymphomas such as BL, Hodgkin lymphoma, diffuse large B-cell lymphoma, plasmablastic lymphoma and post-transplant lymphoproliferative disorder (1–9); rare forms of NK or T-cell lymphomas (10); nasopharyngeal carcinoma and gastric carcinoma. In most EBV-associated malignancies the virus exists in a restricted latency which allows the tumor to escape immune surveillance. Understanding mechanisms of latency restriction will inform approaches to manipulate latency which may generate a more immunogenic tumor and sensitize tumors to T-cell mediated killing.

## 2 EBV infection and establishment of latency

**Figure 1:**
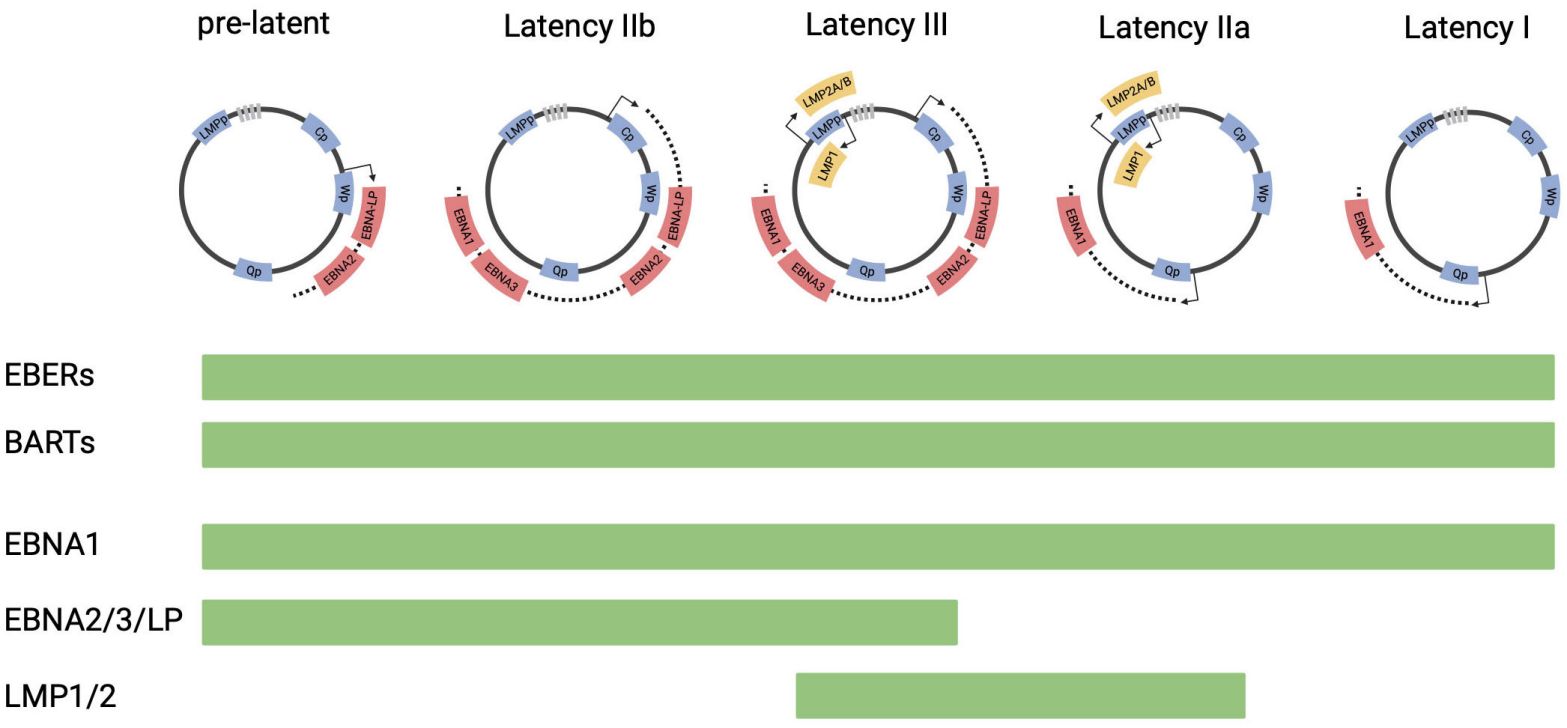
EBV latency programs. This figure depicts the progression of EBV latency gene expression form initial infection to latency I, from left to right. The EBV genome is illustrated in episomal form closed at the terminal repeats (marked by gray boxes). Promoters are shown as blue boxes, including EBNA (Cp, Wp and Qp) and LMP promoters. Coding regions of transcripts are shown as red boxes (EBNAs) and yellow boxes (LMPs). The list of viral genes expressed in each latency state is shown in bar charts below the diagram.

The EBV virion is made up of a 172kB double stranded DNA genome encapsulated by an icosahedral glycoprotein capsid. During the primary infection, orally transmitted EBV can infect epithelial cells and local B cells in the oropharynx, through the binding of the viral capsid glycoprotein gp350 to the surface receptor, CD21 (11–13). Upon infection, the internalized virions translocate to the nucleus, where the linear genome circularizes to form an extrachromosomal episome. The virus then undergoes a series of epigenetically controlled transitions between different latency states (**Figure 1**). In the initial phase, called pre-latency, EBV nuclear antigen 2 (EBNA2) and EBV nuclear antigen leader protein (EBNA-LP) are expressed from the viral W promoter (Wp) (14). In addition, lytic genes, such as *BHRF1*, *BOLF1* and *BPLF1* can be expressed despite the absence of lytic DNA replication (15). As the expression of the lytic genes are downregulated, the expression of EBNA2 and EBNA-LP increases, activating the major upstream viral C promoter (Cp) (16). Cp then drives the expression of all 6 EBNAs (EBNA1, EBNA2, EBNA3A, 3B, 3C and EBNA-LP). This stage is referred to as latency IIb. Through EBNA2-mediated activation of the LMP promoter, EBV infected cells then further transition into latency III program, where the entire panel of latency genes including the EBNAs and LMP1/2 are expressed (17). To evade immune recognition by EBV-specific cytotoxic T-cells, EBV infected cells then downregulate EBNA proteins, resulting in a more restricted form of latency, defined as latency IIa. The latency IIa program, which is observed in germinal center B cells of healthy individuals, is characterized by the expression of LMP1/2 and EBNA 1 from the Qp promoter (18). EBV-infected cells can then downregulate the expression of LMP1/2, expressing only EBNA1 and EBV ncRNAs, including EBERS and BART miRNAs. This state is termed latency I and is often observed in memory B cells. In some instances, no viral proteins are expressed in these cells (Latency 0).

**Figure 2:**
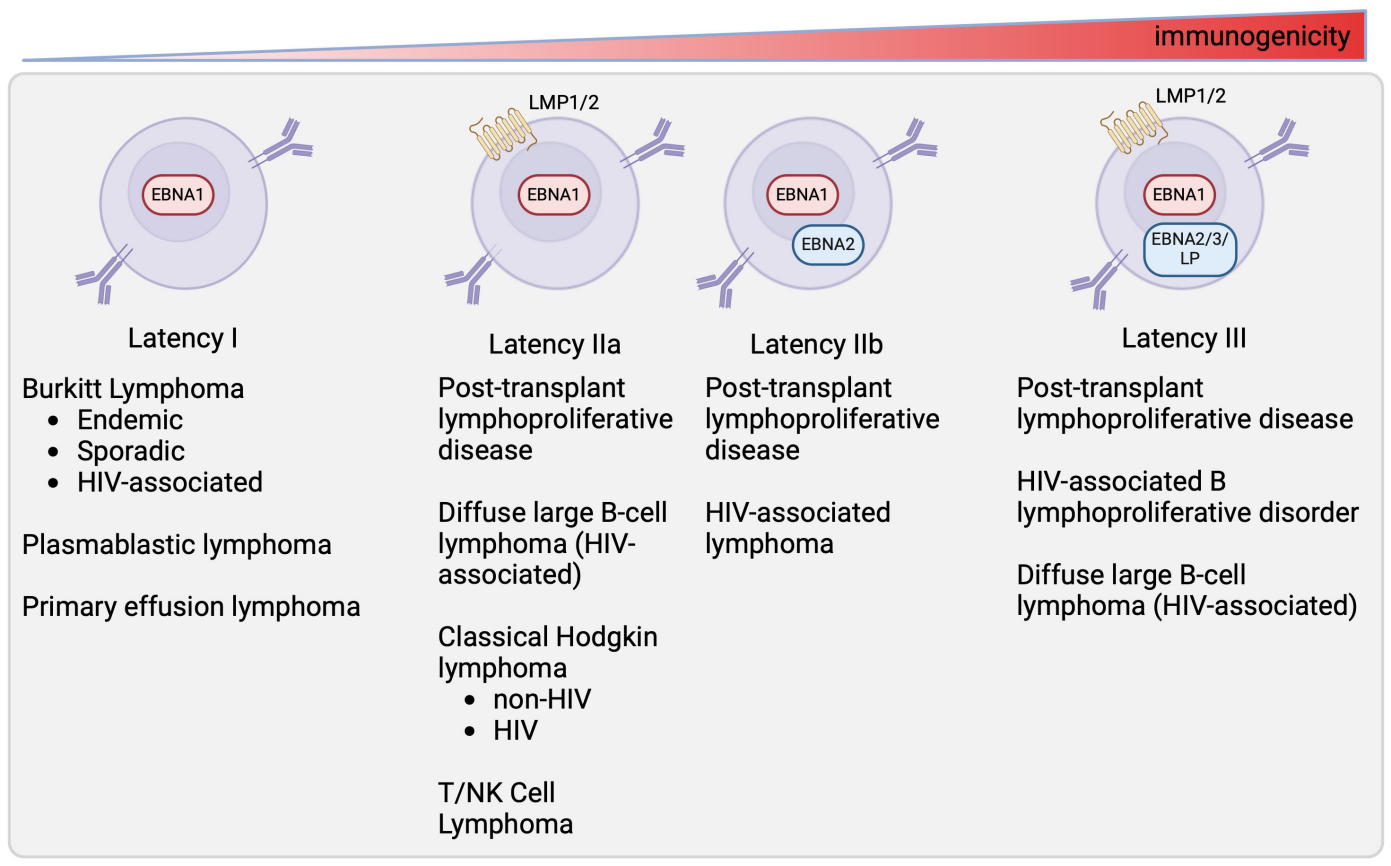
Latency state of different EBV-associated malignancies. This figure depicts latency programs found in different tumors (10, 18–23). The tumors expressing each latency program are listed below.

EBV-associated malignancies adopt specific latency programs based on the tumor type and the status of the host immune system (**Figure 2**). Burkitt lymphoma, plasmablastic lymphoma, primary effusion lymphoma, and gastric carcinoma tumors are characterized by the latency I program, expressing only EBNA1 (24). Latency IIa tumors include EBV-associated Hodgkin lymphoma, NK/T-cell lymphomas, and nasopharyngeal carcinoma (10, 25). Latency III tumors are found in immunocompromised individuals, such as those who have received a solid organ or hematopoietic stem cell transplant or those with HIV. Tumors that can persist in latency III include post-transplant lymphoproliferative disorder (PTLD), and HIV-associated DLBCL (19, 20, 26). Some tumors are heterogeneous in latency patterns. For example, subsets of PTLD and HIV-associated DLBCL, which are typically latency III, can express the latency I or II program and HIV^+^ BL, which is known to be latency I, can express latency II (18–21).

## 3 Epigenetic regulation of EBV latency

The EBV lifecycle in both normal and malignant cells is tightly regulated through epigenetic mechanisms. These mechanisms range from DNA methylation to chromatin modification to three-dimensional genome organization. Improved understanding of the epigenetic control of viral latency may allow us to modulate viral latency programs in EBV-associated malignancies using epigenetic modifying agents.

### 3.1 DNA methylation

DNA methylation of promoter sites, which occurs in CpG-rich segments of DNA, is a key mechanism of epigenetic gene repression in both human and viral genes (27). This process is regulated by a family of DNA methyltransferase (DNMT) enzymes through the transfer of methyl group (CH_3_) to the cytosine nucleotide, forming 5-methylcytosine (5mC) (27). There are three major DNMTs that drive DNA methylation. DNMT1 mainly maintains the DNA methylation pattern during daughter strand DNA synthesis, while DNMT3a and DNMT3b establish new methylation pattern of unmodified DNA. This process can be reversed by the ten-eleven translocation (TET) enzyme, which converts the 5mC to cytosines by oxidizing the 5mC to form 5-hydroxymethylcytosine (5hmC) (28).

In latent EBV infection, the majority of the viral gene promoters are methylated, inhibiting the expression of viral genes which is essential for the maintenance of latency. The level of DNA methylation on Cp and LMP1 promoters in latency I cells is significantly higher than that observed in latency III cells, leading to repression of EBNA 1, 2, 3, LP (from the Cp promoter) and LMP1 (29–31). The Qp promoter remains unmethylated in latency I, allowing for transcription of EBNA1 only. The expression of DNMT1 and DNMT3b is increased in latency I cell lines in comparison to their latency III counterparts (32). The role of DNA methylation in maintaining latency in EBV-associated malignancies is further supported by the studies demonstrating that the DNMT inhibitor, 5-azacytidine can de-repress Cp and LMP transcripts in latency I BL cells (33–35). Importantly, knock-down of DNMT1 in latency I EBV+ BL also induces expression of latency III viral proteins, further highlighting the importance of DNMT1 in maintaining EBV latency (36).

Emerging data also supports a role for *TET2* gene activity in the modulation and maintenance of EBV latency. TET enzymes reverse the effect of DNMTs, resulting in DNA hypomethylation. *TET2* expression is observed in latency III BL cells and LCLs, but not in latency I (37). Depletion of TET2 in latency III cells causes an increase in DNA methylation which corresponds with the decrease in latency III viral gene expression (EBNA2/3A/3B/3C and LMP1/2). The loss of TET2 also results in activation of lytic genes, such as *BZLF1* and *BRLF1*. EBNA2 has been shown to interact with TET2. Inactivation of EBNA2 leads to a corresponding decrease in TET2 mRNA and protein, suggesting that EBNA2 is required for TET2 expression. Co-immunoprecipitation studies in BL cell lines also showed that TET2 interacts with EBNA2, suggesting that these proteins cooperate to reverse DNA methylation regulate EBV latent gene expression (37).

### 3.2 Histone modification

Histone modification is an important post-translational process that plays a key role in regulating gene expression (38, 39). Several types of histone modifications have been described, including acetylation, methylation, ubiquitination and phosphorylation, with acetylation and methylation being the two most well-studied histone modifications. Histone acetylation is typically associated with active gene transcription and histone methylation with gene repression.

Genome-wide ChIP-sequencing studies has revealed the role of histone tail modifications in regulating the latency states of EBV positive cells (40). The epigenetic landscape of viral genome is significantly different in latency I cells in comparison to cells in latency III program, particularly at the transcription start sites for the latency III-specific viral genes (41). The Cp and LMP1/2 promoters are highly enriched for epigenetic marks associated with active transcription (ie H3K9ac, H3K27ac, H4ac and H3K4me3) in latency III cells, but are absent in latency I cells. In contrast, Qp, a promoter that drives EBNA1 transcription in latency I cells and not latency III, is enriched with transcription activation marks (H3K9ac, H4ac, H3K4me3 and H3K4me3) in latency I cells (29, 42). In addition, high levels H3K9me3, an epigenetic mark associated with transcription silencing is observed at the W repeats, Cp and LMP1/2 promoters in latency I cells. In contrast low levels of H3K9me3 are observed across the viral genome in latency III cells (42, 43). Interestingly, both latency I and latency III cells have enriched H3K27me3 at the lytic immediate early promoters (42, 44, 45). H3K27me3 is an epigenetic mark generally associated with gene repression mediated by polycomb repressive complex 1/2 (PRC1/2). As polycomb-associated H3K27me3 has been associated with the regulation of Kaposi’s sarcoma-associated herpesvirus (KSHV) latency, it is possible that PRC1/2 complex has a role in regulating EBV latency program (46, 47).

EBNA2 has been shown to interact with histone acetyltransferases (HATs) such as p300, CREB-binding protein (CBP) and lysine acetyltransferase 2B (KAT2B), and transcription factors such as recombination signal binding protein for immunoglobulin kappa j region (RBP-jK) and PU.1, to modulate both viral and host gene expression (48). This interaction with HATs allows EBNA2 to regulate epigenetic reprogramming of viral and host genome through histone tail modifications. EBNA2 also cooperates with EBNA-LP to remove repressive complexes from promoters, enhancers and matrix-associated deacetylase bodies to activate viral and cellular gene transcription (49).

### 3.3 3D genome organization

Higher order DNA loop structures that control long range DNA interactions are known to exist in EBV and likely play in role in the regulation of viral latency (50). Specifically, a chromatin loop exists between the enhancer OriP and either Qp in latency I or Cp in latency III. Host factors that regulate EBV long rage DNA interactions such as this loop formation include the transcriptional regulator *CTCF* and cohesion subunits, both of which stabilize the interaction between distant DNA sites.

Genome-wide studies have identified CTCF binding sites at several key regulatory elements in the EBV genome (42). The CTCF protein regulates gene transcription by acting as a transcriptional activator, a repressor or an insulator protein by blocking the interaction between enhancers and promoters (51). CTCF also plays an important role in regulating the three-dimensional organization of the genome by promoting long-distance interactions between different DNA regions. CTCF binding sites are found upstream of Qp, Cp and the EBER transcription start site, and downstream of Wp (29). The integrity of CTCF binding sites is critical for the formation and maintenance of the chromatin loop between the OriP and either Qp (in latency I) or Cp (in latency III), which allows for upregulation of their respective viral latency genes.

In addition to CTCF, Poly(ADP-ribose) polymerase 1 (PARP1), a co-factor of CTCF has also been implicated in the regulation of EBV latency and lytic reactivation (52). PARP1 is well characterized in its role in DNA damage, but in recent years, PARP1 has also been implicated in chromatin modification and transcriptional regulation (53–55). PARP1 catalyzes the transfer of poly(ADP-ribose) moiety from nicotinamide adenine dinucleotide (NAD+) onto proteins such as histones and CTCF (52). Poly(ADP-ribosylation of CTCF regulates CTCF’s insulator activity, DNA binding capacity and the ability to form chromatin loops. PARP1 and CTCF colocalize at specific sites throughout the EBV genome (52), stabilizing CTCF binding and maintaining the open chromatin landscape at the active Cp promoter during latency III (52). PARP1 also binds to the BZLF1 lytic switch promoter to restrict EBV reactivation (56).

CTCF can also interact with the cohesin subunits SMC1, SMC3 and RAD21 (57–59). Cohesin plays a crucial role in keeping the sister chromatids together during mitosis and meiosis of dividing cells (60). Additionally, cohesin has also been shown to be crucial for DNA repair, gene expression and genome integrity (61, 62). Loss of cohesin subunits Rad21 and SMC1 results in a complete loss of long-range interaction between OriP and LMP1/LMP2 promoter regions, resulting in an increase in EBV latent gene expression *LMP1/2*. Depletion of cohesion subunits also leads to a modest increase in *BZLF1* expression (40). This suggests that cohesion subunits may also play a role in regulating EBV latency and supports the model that EBV chromatin forms loop formation between OriP and LMP1/2 locus to help maintain viral latent cycle gene expression.

In summary, the repression of the EBV genome in latency is tightly regulated through multiple mechanisms including DNA methylation, histone modification, and higher order chromatin structure. When EBV transitions from latency to lytic reactivation, the repression of lytic programs must be reversed, a process that demonstrates the plasticity of the latent state. Emerging evidence has shown that the lytic viral protein BZLF1 can overcome repression of lytic genes though viral and host interactions. Specifically, BZLF1, can act as a pioneer transcription factor (PTF) that can directly bind viral nucleosomal DNA, recruit chromatin remodelers and enhance the local accessibility of chromatin (63–65). BZLF1 can bind to CpG-methylated motifs on key viral promoters, inducing a loss of polycomb repression and recruit RNA polymerase II to the activated early promoters promoting efficient lytic viral gene expression. Interestingly, DNA methylation is maintained throughout the phase of viral reactivation despite the active transcription of extensively CpG methylated viral genes (64). BZLF1 also imparts changes on host chromatin that may support viral production. Upon lytic activation BZLF1 binds cellular chromatin resulting in reduced chromatin accessibility and decreased chromatin to chromatin interactions in addition to global transcriptional downregulation (65). These cellular changes may support lytic viral production and highlight the profound impact of the EBV on the host cell.

## 4 Cellular therapies targeting EBV latent antigens

Autologous or allogeneic EBV-specific cytotoxic T lymphocytes (EBV-CTLs), which predominantly react against EBNA2/3 and/or LMP1/2A are a promising therapeutic option for EBV-associated lymphomas with the latency II or latency III program. This therapeutic approach has been most extensively studied in the context of post-transplant lymphoproliferative disorder (PTLD). PTLD tumors exhibit type III latency program, expressing the full panel of viral protein and genes. These viral proteins are highly immunogenic and are recognized by cytotoxic T cells, making them ideal targets for T cell immunotherapy. In 1995, Rooney and colleagues infused donor-derived EBV-CTLs in 10 hematopoietic stem cell transplant (HSCT) recipients as either treatment of EBV reactivation/proliferation (n=3) or as prophylaxis (n=7) (66). EBV DNA concentration of all three patients with EBV reactivation returned to the basal range within 3-4 weeks of immunotherapy. One patient also showed resolution of immunoblastic lymphoma. With this promising result, a follow-up study was carried out to examine the persistence of EBV-specific CTLs. Fourteen patients who were at high risk to develop EBV associated PTLD following T cell depleted bone marrow transplantation received donor-derived EBV-specific CTLs. The transferred CTLs not only reconstituted the patients’ immune response to EBV, retaining their ability to respond to viral stimulation *in vivo*, but also persisted after 18 months (67). The efficacy of donor-derived EBV-CTLs has been demonstrated in a number of case series among patients with PTLD after HSCT with overall response rates ranging from 29-73% (68–70). Autologous EBV-CTLs have also been demonstrated to induce complete responses in patients with PTLD after solid organ transplant (SOT) (71–73). More recently “off-the-shelf” EBV-CTLs have been generated from healthy HSCT donors and investigated as treatment for PTLD in HSCT and SOT recipients (74, 75). Among 46 recipients, the overall response rate was 68% and 54% for HSCT and SOT recipients respectively. Complete responses were observed in 19/33 HSCT recipients and 2/13 SOT recipients (75). This approach is now being studied in an ongoing phase III trial (clincaltrial.gov NCT03394365).

EBV-CTLs have also demonstrated efficacy in EBV+ Hodgkin lymphoma tumors which express the latency II program (HL) (76, 77). Bollard et al. evaluated EBV-CTL treatment among 14 patients with relapsed HL who either had measurable disease or were at high risk of recurrence. EBV-CTLs were generated using patient-derived EBV-transformed LCLs (78). These LCLs predominantly enrich for the T cells specific for the early lytic cycle transactivators BZLF1 and BMLF1 and for the latency III associated EBNAs (EBNA3A/3B/3C). Even though these antigens are not expressed in the tumors of HL, these LCLs also reactivate T cells specific for LMP2, which is expressed by the tumor. The team hypothesized that LMP2 specific CTLs would expand *in-vivo* upon exposure to tumor (78). Among patients with measurable tumor, 2/8 patients showed complete response, 5 had stable disease and 1had a partial response. In a follow up study, EBV-CTLs were engineered with a dominant negative TGF-beta receptor type II (DNRII) to avoid immune evasion (79). DNRII T-cells specific for LMP1 and LMP2 were evaluated in a dose escalation study in 8 patients with EBV+ HL. The cells safely expanded and persisted *in-vivo* and a durable complete response was observed in 4/7 evaluable patients (79). Overall, these studies demonstrate the robust and effective T-cell response to latency II and III EBV antigens.

## 5 Latency switching to sensitize latency I tumors to latency II/III directed cellular therapy

Given the success in EBV-directed immunotherapy in cancers with latency II and latency III program and earlier studies indicating that latency switch can be induced through pharmacological interventions *in-vitro*, our team hypothesized that pharmacologic conversion of latency I tumors to latency II/III could be achieved *in-vivo* and would sensitize latency I tumors to EBV-directed immunotherapy. To investigate this we performed a high-throughput compound screen that included 441 cancer compounds to identify agents capable of inducing latency II/III antigens in latency I EBV^+^ Burkitt lymphoma (34). Top hits from this screen included epigenetic modifiers such as the hypomethylating agent 5-azacytidine, the histone deacetylase inhibitor panobinostat, and inhibitors of the histone methyl transferase Enhancer of zeste homolog 2 (EZH2). Other hits included proteosome inhibitors such as bortezomib and agents targeting cell cycle/DNA damage such as cytarabine, doxorubicin, and pralatrexate. In a focused epigenetic screen the DNMT1 inhibitor decitabine emerged as the most potent inducer of latency II/III antigens in BL. Our findings were further supported by a CRISPR/Cas9 screen which revelated that DNA methyltransferase I (DNMT1) and its partner ubiquitin-like with PHD and RING finger domain containing protein (UHRF1) are essential to the repression of latency III in BL (36).

Decitabine treatment of latency I BL results in hypomethylation at the LMP1 and Cp promoters and induces expression EBNA2 and LMP1 *in-vitro* and *in-vivo* in xenograft models of latency I EBV^+^ BL. The combination of decitabine followed by EBV-CTLs in xenograft models resulted in homing of EBV-CTLs to the tumor and tumor regressions (34). This work demonstrates the key role of promoter methylation in the latency restriction and suggests that pharmacologic induction of latency II/III with hypomethylating agents may sensitize tumors to T-cell mediated killing. Importantly, latency conversion with decitabine was only observed in a subset of cells (20-55%). It will be essential to identify approaches for enhanced conversion to effectively deploy this therapeutic approach.

## 6 Targeting EBV lytic proteins to treat EBV-associated malignancies

In addition to targeting the EBV latency program for therapy, an alternative approach is to target the EBV lytic proteins. Conversion from the latent to the lytic state could sensitize cells to anti-viral agents such as the deoxynucleoside analog ganciclovir (GCV) and/or T-cells directed against lytic antigens. Anti-viral agents directed against EBV, such as GCV are ineffective in EBV-associated malignancies because these drugs require monophosphylation by EBV- thymidine kinase, an enzyme that is not expressed in the latent state (80–84). Early studies examining lytic induction followed by antiviral therapy utilized arginine butyrate to induce lytic replication. Arginine butyrate treatment of EBV^+^ immunoblastic non-Hodgkin lymphoma (NHL) cells was found to induce the expression of EBV thymidine kinase and sensitize cells to GCV resulting in inhibition of cell proliferation and cell death (85). A phase I/II clinical trial for the combination of arginine butyrate and GCV showed that the treatment combination was well-tolerated and resulted in complete or partial clinical responses in 10 out of 15 patients with EBV-associated lymphoid malignancies which had been previously refractory to all conventional therapies (86)**.**

In addition to arginine butyrate, epigenetic modifiers (5-azacytadine and romidepsin) as well as other compounds (trichostatin A and valproic acid) have also been shown to induce the expression of lytic genes in EBV^+^ tumors and enhance the cytotoxic effect of GCV (80–82). Recently, a phase Ib/II clinical trial was performed to examine the effect of nanatinostat, a class-I specific HDAC inhibitor in combination with valganciclovir (VGCV), an orally bioavailable prodrug of GCV in patients with EBV^+^ lymphoma (NCT03397706) (87). The overall response rate was 40% (n=17/43), with eight patients demonstrating a complete response. Due to the promising result of this trial, a phase II study (NCT05011058) for the combination of nanatinostat and VGCV is currently ongoing.

Despite the promising effect of lytic induction and anti-viral therapy, the efficacy of this treatment strategy greatly depends on the efficiency of lytic induction, as the cytotoxicity effect of anti-viral therapy such as GCV relies on the expression of viral lytic proteins. Thus, the development of more specific and potent lytic-inducing agents is crucial to improve this therapy strategy.

## 7 Conclusion

EBV-associated lymphomas are responsible for a significant health burden worldwide. The predominant mechanism that allows these tumors to evade the host immune response to EBV and persist is the adoption of restricted forms of viral latency. Recent studies have elucidated the many complex mechanisms by which the virus is able to maintain restricted latency including DNA methylation at key promoter regions, chromatin modifications, and 3D chromosome organization. Still, there remain gaps in knowledge about how the viral latency programs are established and when this process occurs, whether during early B cell development or in the germinal center, where most EBV^+^ B-cell lymphomas arise. Improved understanding of these processes will inform strategies to modulate viral latency.

Our work has shown that hypomethylation of EBV^+^ latency I BL with decitabine induces the expression of immunogenic viral antigens, sensitizing these tumors to T cell mediated killing, suggesting that latency conversion may be a feasible therapeutic option for EBV-associated malignancies with restricted latency. A limitation to this approach is that latency switch is not induced in all cells which may ultimately lead to immune escape and resistance. Further studies will be required to understand the role of DNMT1 in regulating the EBV latency program and potential combination therapies that may enhance latency switching and/or enhance the immune response to a latency-converted tumor. Potential combinations include: 1) hypomethylating agents in combination with other agents known to induce latency conversion such as HDAC inhibitors, EZH2 inhibitors, or agents that target DNA damage or cell cycle; 2) hypomethylating agents in combination with agents that to combat compensatory epigenetic mechanisms that may be responsible for resistance; 3) hypomethylating agents in combination with immunomodulatory agents that may augment the immune response to latent antigens, such as bispecific T-cell engagers or checkpoint inhibitors. Overall, an expanded understanding how the latency program is regulated will be crucial for the development of future viral and host epigenetic directed therapies that could be applied to all EBV-associated malignancies of restricted latency.

## Author contributions

IK: Writing – original draft, Writing – review & editing. LG-R: Writing – original draft, Writing – review & editing.

## References

[1] Anwar N Kingma DW Bloch AR Mourad M Raffeld M Franklin J. The investigation of Epstein-Barr viral sequences in 41 cases of Burkitt’s lymphoma from Egypt: epidemiologic correlations. Cancer. (1995) 76:1245–52. doi: 10.1002/1097-0142(19951001)76:7<1245::aid-cncr2820760723>3.0.co;2-d 8630905

[2] Klumb CE Hassan R De Oliveira DE De Resende LM Carrico MK De Almeida Dobbin J. Geographic variation in Epstein-Barr virus-associated Burkitt’s lymphoma in children from Brazil. Int J Cancer. (2004) 108:66–70. doi: 10.1002/ijc.11443. 14618617

[3] Massini G Siemer D Hohaus S. EBV in hodgkin lymphoma. Mediterr J Hematol Infect Dis. (2009) 1:e2009013. doi: 10.4084/mjhid. 21416003 PMC3033177

[4] Shibata D Weiss LM. Epstein-Barr virus-associated gastric adenocarcinoma. Am J Pathol. (1992) 140:769–74. PMC1886378 1314023

[5] Tsao SW Tsang CM Lo KW. Epstein-Barr virus infection and nasopharyngeal carcinoma. Philos Trans R Soc Lond B Biol Sci. (2017) 372(1732). doi: 10.1098/rstb.2016.0270. PMC5597737 28893937

[6] Healy JA Dave SS. The role of EBV in the pathogenesis of diffuse large B cell lymphoma. Curr Top Microbiol Immunol. (2015) 390:315–37. doi: 10.1007/978-3-319-22822-8_13 26424652

[7] Kato S Yamashita D Nakamura S. Nodal EBV+ cytotoxic T-cell lymphoma: A literature review based on the 2017 WHO classification. J Clin Exp Hematop. (2020) 60:30–6. doi: 10.3960/jslrt.20001. PMC7337268 32565530

[8] List AF Greco FA Vogler LB. Lymphoproliferative diseases in immunocompromised hosts: the role of Epstein-Barr virus. J Clin Oncol. (1987) 5:1673–89. doi: 10.1200/JCO.1987.5.10.1673. 2821199

[9] Cesarman E. Gammaherpesviruses and lymphoproliferative disorders. Annu Rev Pathol. (2014) 9:349–72. doi: 10.1146/annurev-pathol-012513-104656. 24111911

[10] Quintanilla-Martinez L Swerdlow SH Tousseyn T Barrionuevo C Nakamura S Jaffe ES. New concepts in EBV-associated B, T, and NK cell lymphoproliferative disorders. Virchows Archiv. (2023) 482:227–44. doi: 10.1007/s00428-022-03414-4. PMC9852222 36216980

[11] Fingeroth JD Clabby ML Strominger JD. Characterization of a T-lymphocyte Epstein-Barr virus/C3d receptor (CD21). J Virol. (1988) 62:1442–7. doi: 10.1128/jvi.62.4.1442-1447.1988. PMC253159 2831405

[12] Fingeroth JD Weis JJ Tedder TF Strominger JL Biro PA Fearon DT. Epstein-Barr virus receptor of human B lymphocytes is the C3d receptor CR2. Proc Natl Acad Sci U.S.A. (1984) 81:4510–4. doi: 10.1073/pnas.81.14.4510. PMC345620 6087328

[13] Tanner J Weis J Fearon D Whang Y Kieff E. Epstein-Barr virus gp350/220 binding to the B lymphocyte C3d receptor mediates adsorption, capping, and endocytosis. Cell. (1987) 50:203–13. doi: 10.1016/0092-8674(87)90216-9. 3036369

[14] Woisetschlaeger M Strominger JL Speck SH. Mutually exclusive use of viral promoters in Epstein-Barr virus latently infected lymphocytes. Proc Natl Acad Sci U.S.A. (1989) 86:6498–502. doi: 10.1073/pnas.86.17.6498. PMC297871 2549539

[15] Wang C Li D Zhang L Jiang S Liang J Narita Y. RNA sequencing analyses of gene expression during epstein-barr virus infection of primary B lymphocytes. J Virol. (2019) 93(13). doi: 10.1128/JVI.00226-19. PMC6580941 31019051

[16] Alfieri C Birkenbach M Kieff E. Early events in Epstein-Barr virus infection of human B lymphocytes. Virology. (1991) 181:595–608. doi: 10.1016/0042-6822(91)90893-G. 1849678

[17] Kieff E. Epstein-Barr virus and its replication. Field’s Virol. (1996) 1996:2348–96.

[18] Price AM Luftig MA. To be or not IIb: a multi-step process for Epstein-Barr virus latency establishment and consequences for B cell tumorigenesis. PloS Pathog. (2015) 11:e1004656. doi: 10.1371/journal.ppat.1004656. 25790223 PMC4366242

[19] Arvey A Ojesina AI Pedamallu CS Ballon G Jung J Duke F. The tumor virus landscape of AIDS-related lymphomas. Blood. (2015) 125:e14–22. doi: 10.1182/blood-2014-11-599951. PMC4432014 25827832

[20] Brink AA Dukers DF van den Brule AJ Oudejans JJ Middeldorp JM Meijer CJ. Presence of Epstein-Barr virus latency type III at the single cell level in post-transplantation lymphoproliferative disorders and AIDS related lymphomas. J Clin Pathol. (1997) 50:911–8. doi: 10.1136/jcp.50.11.911. PMC500314 9462239

[21] Oudejans JJ Jiwa M van den Brule AJ Grasser FA Horstman A Vos W. Detection of heterogeneous Epstein-Barr virus gene expression patterns within individual post-transplantation lymphoproliferative disorders. Am J Pathol. (1995) 147:923–33. PMC1871006 7573368

[22] Doyle MG Catovsky D Crawford DH. Infection of leukaemic B lymphocytes by Epstein Barr virus. Leukemia. (1993) 7:1858–64. 8231253

[23] Kurth J Spieker T Wustrow J Strickler GJ Hansmann LM Rajewsky K. EBV-infected B cells in infectious mononucleosis: viral strategies for spreading in the B cell compartment and establishing latency. Immunity. (2000) 13:485–95. doi: 10.1016/S1074-7613(00)00048-0. 11070167

[24] Rowe M Rowe DT Gregory CD Young LS Farrell PJ Rupani H. Differences in B cell growth phenotype reflect novel patterns of Epstein-Barr virus latent gene expression in Burkitt’s lymphoma cells. EMBO J. (1987) 6:2743–51. doi: 10.1002/embj.1987.6.issue-9. PMC553698 2824192

[25] Thorley-Lawson DA Gross A. Persistence of the Epstein-Barr virus and the origins of associated lymphomas. N Engl J Med. (2004) 350:1328–37. doi: 10.1056/NEJMra032015. 15044644

[26] Nalesnik MA. Clinicopathologic characteristics of post-transplant lymphoproliferative disorders. Recent Results Cancer Res. (2002) 159:9–18. doi: 10.1007/978-3-642-56352-2_2 11785849

[27] Moore LD Le T Fan G. DNA methylation and its basic function. Neuropsychopharmacology. (2013) 38:23–38. doi: 10.1038/npp.2012.112. 22781841 PMC3521964

[28] Rasmussen KD Helin K. Role of TET enzymes in DNA methylation, development, and cancer. Genes Dev. (2016) 30:733–50. doi: 10.1101/gad.276568.115. PMC4826392 27036965

[29] Tempera I Wiedmer A Dheekollu J Lieberman PM. CTCF prevents the epigenetic drift of EBV latency promoter Qp. PloS Pathog. (2010) 6:e1001048. doi: 10.1371/journal.ppat.1001048. 20730088 PMC2921154

[30] Takacs M Salamon D Myohanen S Li H Segesdi J Ujvari D. Epigenetics of latent Epstein-Barr virus genomes: high resolution methylation analysis of the bidirectional promoter region of latent membrane protein 1 and 2B genes. Biol Chem. (2001) 382:699–705. doi: 10.1515/BC.2001.083. 11405234

[31] Falk KI Szekely L Aleman A Ernberg I. Specific methylation patterns in two control regions of Epstein-Barr virus latency: the LMP-1-coding upstream regulatory region and an origin of DNA replication (oriP). J Virol. (1998) 72:2969–74. doi: 10.1128/JVI.72.4.2969-2974.1998. PMC109743 9525618

[32] Hughes DJ Marendy EM Dickerson CA Yetming KD Sample CE Sample JT. Contributions of CTCF and DNA methyltransferases DNMT1 and DNMT3B to Epstein-Barr virus restricted latency. J Virol. (2012) 86:1034–45. doi: 10.1128/JVI.05923-11. PMC3255836 22072770

[33] Rao SP Rechsteiner MP Berger C Sigrist JA Nadal D Bernasconi M. Zebularine reactivates silenced E-cadherin but unlike 5-Azacytidine does not induce switching from latent to lytic Epstein-Barr virus infection in Burkitt’s lymphoma Akata cells. Mol Cancer. (2007) 6:3. doi: 10.1186/1476-4598-6-3. 17214905 PMC1781464

[34] Dalton T Doubrovina E Pankov D Reynolds R Scholze H Selvakumar A. Epigenetic reprogramming sensitizes immunologically silent EBV+ lymphomas to virus-directed immunotherapy. Blood. (2020) 135:1870–81. doi: 10.1182/blood.2019004126. PMC7243148 32157281

[35] Masucci MG Contreras-Salazar B Ragnar E Falk K Minarovits J Ernberg I. 5-Azacytidine up regulates the expression of Epstein-Barr virus nuclear antigen 2 (EBNA-2) through EBNA-6 and latent membrane protein in the Burkitt’s lymphoma line rael. J Virol. (1989) 63:3135–41. doi: 10.1128/jvi.63.7.3135-3141.1989. PMC250871 2470924

[36] Guo R Zhang Y Teng M Jiang C Schineller M Zhao B. DNA methylation enzymes and PRC1 restrict B-cell Epstein-Barr virus oncoprotein expression. Nat Microbiol. (2020) 5:1051–63. doi: 10.1038/s41564-020-0724-y. PMC7462085 32424339

[37] Lu F Wiedmer A Martin KA Wickramasinghe P Kossenkov AV Lieberman PM. Coordinate regulation of TET2 and EBNA2 controls the DNA methylation state of latent epstein-barr virus. J Virol. (2017) 91(20). doi: 10.1128/JVI.00804-17. PMC5625499 28794029

[38] Liu R Wu J Guo H Yao W Li S Lu Y. Post-translational modifications of histones: Mechanisms, biological functions, and therapeutic targets. MedComm (2020). (2023) 4:e292. doi: 10.1002/mco2.292 37220590 PMC10200003

[39] Alaskhar Alhamwe B Khalaila R Wolf J von Bulow V Harb H Alhamdan F. Histone modifications and their role in epigenetics of atopy and allergic diseases. Allergy Asthma Clin Immunol. (2018) 14:39. doi: 10.1186/s13223-018-0259-4. 29796022 PMC5966915

[40] Arvey A Tempera I Tsai K Chen HS Tikhmyanova N Klichinsky M. An atlas of the Epstein-Barr virus transcriptome and epigenome reveals host-virus regulatory interactions. Cell Host Microbe. (2012) 12:233–45. doi: 10.1016/j.chom.2012.06.008. PMC3424516 22901543

[41] Gerle B Koroknai A Fejer G Bakos A Banati F Szenthe K. Acetylated histone H3 and H4 mark the upregulated LMP2A promoter of Epstein-Barr virus in lymphoid cells. J Virol. (2007) 81:13242–7. doi: 10.1128/JVI.01396-07. PMC2169097 17898065

[42] Day L Chau CM Nebozhyn M Rennekamp AJ Showe M Lieberman PM. Chromatin profiling of Epstein-Barr virus latency control region. J Virol. (2007) 81:6389–401. doi: 10.1128/JVI.02172-06. PMC1900095 17409162

[43] Arvey A Tempera I Lieberman PM. Interpreting the Epstein-Barr Virus (EBV) epigenome using high-throughput data. Viruses. (2013) 5:1042–54. doi: 10.3390/v5041042. PMC3705264 23549386

[44] Murata T Kondo Y Sugimoto A Kawashima D Saito S Isomura H. Epigenetic histone modification of Epstein-Barr virus BZLF1 promoter during latency and reactivation in Raji cells. J Virol. (2012) 86:4752–61. doi: 10.1128/JVI.06768-11. PMC3347330 22357272

[45] Ramasubramanyan S Osborn K Flower K Sinclair AJ. Dynamic chromatin environment of key lytic cycle regulatory regions of the Epstein-Barr virus genome. J Virol. (2012) 86:1809–19. doi: 10.1128/JVI.06334-11. PMC3264371 22090141

[46] Toth Z Brulois K Jung JU. The chromatin landscape of Kaposi’s sarcoma-associated herpesvirus. Viruses. (2013) 5:1346–73. doi: 10.3390/v5051346. PMC3712311 23698402

[47] Toth Z Maglinte DT Lee SH Lee HR Wong LY Brulois KF. Epigenetic analysis of KSHV latent and lytic genomes. PloS Pathog. (2010) 6:e1001013. doi: 10.1371/journal.ppat.1001013. 20661424 PMC2908616

[48] Wang L Grossman SR Kieff E. Epstein-Barr virus nuclear protein 2 interacts with p300, CBP, and PCAF histone acetyltransferases in activation of the LMP1 promoter. Proc Natl Acad Sci U.S.A. (2000) 97:430–5. doi: 10.1073/pnas.97.1.430. PMC26680 10618435

[49] Portal D Zhou H Zhao B Kharchenko PV Lowry E Wong L. Epstein-Barr virus nuclear antigen leader protein localizes to promoters and enhancers with cell transcription factors and EBNA2. Proc Natl Acad Sci U.S.A. (2013) 110:18537–42. doi: 10.1073/pnas.1317608110. PMC3832032 24167291

[50] Tempera I Klichinsky M Lieberman PM. EBV latency types adopt alternative chromatin conformations. PloS Pathog. (2011) 7:e1002180. doi: 10.1371/journal.ppat.1002180. 21829357 PMC3145795

[51] Kim S Yu NK Kaang BK. CTCF as a multifunctional protein in genome regulation and gene expression. Exp Mol Med. (2015) 47:e166. doi: 10.1038/emm.2015.33. 26045254 PMC4491725

[52] Lupey-Green LN Caruso LB Madzo J Martin KA Tan Y Hulse M. PARP1 stabilizes CTCF binding and chromatin structure to maintain epstein-barr virus latency type. J Virol. (2018) 92(18). doi: 10.1128/JVI.00755-18. PMC6146685 29976663

[53] Wei H Yu X. Functions of PARylation in DNA damage repair pathways. Genomics Proteomics Bioinf. (2016) 14:131–9. doi: 10.1016/j.gpb.2016.05.001. PMC4936651 27240471

[54] Martin KA Cesaroni M Denny MF Lupey LN Tempera I. Global transcriptome analysis reveals that poly(ADP-ribose) polymerase 1 regulates gene expression through EZH2. Mol Cell Biol. (2015) 35:3934–44. doi: 10.1128/MCB.00635-15. PMC4628063 26370511

[55] Zong W Gong Y Sun W Li T Wang ZQ. PARP1: liaison of chromatin remodeling and transcription. Cancers (Basel). (2022) 14(17). doi: 10.3390/cancers14174162. PMC9454564 36077699

[56] Lupey-Green LN Moquin SA Martin KA McDevitt SM Hulse M Caruso LB. PARP1 restricts Epstein Barr Virus lytic reactivation by binding the BZLF1 promoter. Virology. (2017) 507:220–30. doi: 10.1016/j.virol.2017.04.006. PMC5521201 28456021

[57] Rubio ED Reiss DJ Welcsh PL Disteche CM Filippova GN Baliga NS. CTCF physically links cohesin to chromatin. Proc Natl Acad Sci U.S.A. (2008) 105:8309–14. doi: 10.1073/pnas.0801273105. PMC2448833 18550811

[58] Wendt KS Yoshida K Itoh T Bando M Koch B Schirghuber E. Cohesin mediates transcriptional insulation by CCCTC-binding factor. Nature. (2008) 451:796–801. doi: 10.1038/nature06634. 18235444

[59] Stedman W Kang H Lin S Kissil JL Bartolomei MS Lieberman PM. Cohesins localize with CTCF at the KSHV latency control region and at cellular c-myc and H19/Igf2 insulators. EMBO J. (2008) 27:654–66. doi: 10.1038/emboj.2008.1. PMC2262040 18219272

[60] Michaelis C Ciosk R Nasmyth K. Cohesins: chromosomal proteins that prevent premature separation of sister chromatids. Cell. (1997) 91:35–45. doi: 10.1016/S0092-8674(01)80007-6. 9335333

[61] Litwin I Pilarczyk E Wysocki R. The emerging role of cohesin in the DNA damage response. Genes (Basel). (2018) 9(12). doi: 10.3390/genes9120581. PMC6316000 30487431

[62] Horsfield JA. Full circle: a brief history of cohesin and the regulation of gene expression. FEBS J. (2023) 290:1670–87. doi: 10.1111/febs.16362. 35048511

[63] Schaeffner M Mrozek-Gorska P Buschle A Woellmer A Tagawa T Cernilogar FM. BZLF1 interacts with chromatin remodelers promoting escape from latent infections with EBV. Life Sci Alliance. (2019) 2(2). doi: 10.26508/lsa.201800108. PMC6441497 30926617

[64] Woellmer A Arteaga-Salas JM Hammerschmidt W. BZLF1 governs CpG-methylated chromatin of Epstein-Barr Virus reversing epigenetic repression. PloS Pathog. (2012) 8:e1002902. doi: 10.1371/journal.ppat.1002902. 22969425 PMC3435241

[65] Bernaudat F Gustems M Gunther J Oliva MF Buschle A Gobel C. Structural basis of DNA methylation-dependent site selectivity of the Epstein-Barr virus lytic switch protein ZEBRA/Zta/BZLF1. Nucleic Acids Res. (2022) 50:490–511. doi: 10.1093/nar/gkab1183. 34893887 PMC8754650

[66] Rooney CM Smith CA Ng CY Loftin S Li C Krance RA. Use of gene-modified virus-specific T lymphocytes to control Epstein-Barr-virus-related lymphoproliferation. Lancet. (1995) 345:9–13. doi: 10.1016/S0140-6736(95)91150-2. 7799740

[67] Heslop HE Ng CY Li C Smith CA Loftin SK Krance RA. Long-term restoration of immunity against Epstein-Barr virus infection by adoptive transfer of gene-modified virus-specific T lymphocytes. Nat Med. (1996) 2:551–5. doi: 10.1038/nm0596-551. 8616714

[68] Doubrovina E Oflaz-Sozmen B Prockop SE Kernan NA Abramson S Teruya-Feldstein J. Adoptive immunotherapy with unselected or EBV-specific T cells for biopsy-proven EBV+ lymphomas after allogeneic hematopoietic cell transplantation. Blood. (2012) 119:2644–56. doi: 10.1182/blood-2011-08-371971. PMC3311278 22138512

[69] Icheva V Kayser S Wolff D Tuve S Kyzirakos C Bethge W. Adoptive transfer of epstein-barr virus (EBV) nuclear antigen 1-specific t cells as treatment for EBV reactivation and lymphoproliferative disorders after allogeneic stem-cell transplantation. J Clin Oncol. (2013) 31:39–48. doi: 10.1200/JCO.2011.39.8495. 23169501

[70] McLaughlin LP Rouce R Gottschalk S Torrano V Carrum G Wu MF. EBV/LMP-specific T cells maintain remissions of T- and B-cell EBV lymphomas after allogeneic bone marrow transplantation. Blood. (2018) 132:2351–61. doi: 10.1182/blood-2018-07-863654. PMC6265652 30262660

[71] Savoldo B Goss JA Hammer MM Zhang L Lopez T Gee AP. Treatment of solid organ transplant recipients with autologous Epstein Barr virus-specific cytotoxic T lymphocytes (CTLs). Blood. (2006) 108:2942–9. doi: 10.1182/blood-2006-05-021782. PMC1895521 16835376

[72] Khanna R Bell S Sherritt M Galbraith A Burrows SR Rafter L. Activation and adoptive transfer of Epstein-Barr virus-specific cytotoxic T cells in solid organ transplant patients with posttransplant lymphoproliferative disease. Proc Natl Acad Sci U.S.A. (1999) 96:10391–6. doi: 10.1073/pnas.96.18.10391. PMC17898 10468618

[73] Comoli P Maccario R Locatelli F Valente U Basso S Garaventa A. Treatment of EBV-related post-renal transplant lymphoproliferative disease with a tailored regimen including EBV-specific T cells. Am J Transplant. (2005) 5:1415–22. doi: 10.1111/j.1600-6143.2005.00854.x. 15888049

[74] Barker JN Doubrovina E Sauter C Jaroscak JJ Perales MA Doubrovin M. Successful treatment of EBV-associated posttransplantation lymphoma after cord blood transplantation using third-party EBV-specific cytotoxic T lymphocytes. Blood. (2010) 116:5045–9. doi: 10.1182/blood-2010-04-281873. PMC3012598 20826724

[75] Prockop S Doubrovina E Suser S Heller G Barker J Dahi P. Off-the-shelf EBV-specific T cell immunotherapy for rituximab-refractory EBV-associated lymphoma following transplantation. J Clin Invest. (2020) 130:733–47. doi: 10.1172/JCI121127. PMC6994129 31689242

[76] Lucas KG Salzman D Garcia A Sun Q. Adoptive immunotherapy with allogeneic Epstein–Barr virus (EBV)-specific cytotoxic T-lymphocytes for recurrent, EBV-positive Hodgkin disease. Cancer. (2004) 100:1892–901. doi: 10.1002/cncr.20188. 15112270

[77] Bollard CM Gottschalk S Leen AM Weiss H Straathof KC Carrum G. Complete responses of relapsed lymphoma following genetic modification of tumor-antigen presenting cells and T-lymphocyte transfer. Blood. (2007) 110:2838–45. doi: 10.1182/blood-2007-05-091280. PMC2018666 17609424

[78] Bollard CM Aguilar L Straathof KC Gahn B Huls MH Rousseau A. Cytotoxic T lymphocyte therapy for Epstein-Barr virus+ Hodgkin’s disease. J Exp Med. (2004) 200:1623–33. doi: 10.1084/jem.20040890. PMC2211993 15611290

[79] Bollard CM Tripic T Cruz CR Dotti G Gottschalk S Torrano V. Tumor-specific T-cells engineered to overcome tumor immune evasion induce clinical responses in patients with relapsed hodgkin lymphoma. J Clin Oncol. (2018) 36:1128–39. doi: 10.1200/JCO.2017.74.3179. PMC5891126 29315015

[80] Wildeman MA Novalic Z Verkuijlen SA Juwana H Huitema AD Tan IB. Cytolytic virus activation therapy for Epstein-Barr virus-driven tumors. Clin Cancer Res. (2012) 18:5061–70. doi: 10.1158/1078-0432.CCR-12-0574. 22761471

[81] Hui KF Cheung AK Choi CK Yeung PL Middeldorp JM Lung ML. Inhibition of class I histone deacetylases by romidepsin potently induces Epstein-Barr virus lytic cycle and mediates enhanced cell death with ganciclovir. Int J Cancer. (2016) 138:125–36. doi: 10.1002/ijc.29698. 26205347

[82] Jung EJ Lee YM Lee BL Chang MS Kim WH. Lytic induction and apoptosis of Epstein-Barr virus-associated gastric cancer cell line with epigenetic modifiers and ganciclovir. Cancer Lett. (2007) 247:77–83. doi: 10.1016/j.canlet.2006.03.022. 16647201

[83] Ghosh SK Forman LW Akinsheye I Perrine SP Faller DV. Short, discontinuous exposure to butyrate effectively sensitizes latently EBV-infected lymphoma cells to nucleoside analogue antiviral agents. Blood Cells Mol Dis. (2007) 38:57–65. doi: 10.1016/j.bcmd.2006.10.008. 17161633 PMC1829174

[84] Ghosh SK Perrine SP Williams RM Faller DV. Histone deacetylase inhibitors are potent inducers of gene expression in latent EBV and sensitize lymphoma cells to nucleoside antiviral agents. Blood. (2012) 119:1008–17. doi: 10.1182/blood-2011-06-362434. PMC3271713 22160379

[85] Mentzer SJ Fingeroth J Reilly JJ Perrine SP Faller DV. Arginine butyrate-induced susceptibility to ganciclovir in an Epstein-Barr-virus-associated lymphoma. Blood Cells Mol Dis. (1998) 24:114–23. doi: 10.1006/bcmd.1998.0178. 9628848

[86] Perrine SP Hermine O Small T Suarez F O'Reilly R Boulad F. A phase 1/2 trial of arginine butyrate and ganciclovir in patients with Epstein-Barr virus-associated lymphoid Malignancies. Blood. (2007) 109:2571–8. doi: 10.1182/blood-2006-01-024703 PMC1852196 17119113

[87] Haverkos B Alpdogan O Baiocchi R Brammer JE Feldman TA Capra M. Targeted therapy with nanatinostat and valganciclovir in recurrent EBV-positive lymphoid Malignancies: a phase 1b/2 study. Blood Adv. (2023) 7:6339–50. doi: 10.1182/bloodadvances.2023010330. PMC10587711 37530631

